# Glycerol kinase 2 is essential for proper arrangement of crescent-like mitochondria to form the mitochondrial sheath during mouse spermatogenesis

**DOI:** 10.1262/jrd.2018-136

**Published:** 2019-01-21

**Authors:** Keisuke SHIMADA, Hirotaka KATO, Haruhiko MIYATA, Masahito IKAWA

**Affiliations:** 1) Research Institute for Microbial Diseases, Osaka University, Osaka 565-0871, Japan; 2) The Institute of Medical Science, The University of Tokyo, Tokyo 108-8639, Japan

**Keywords:** Glycerol kinase, Male infertility, Mitochondrial sheath formation, Spermatogenesis, Sperm mitochondria

## Abstract

The mitochondrial sheath is composed of mitochondria that coil tightly around the midpiece of sperm flagellum. These mitochondria are recruited from the cytoplasm to the flagellum late in spermatogenesis. Initially, recruited mitochondria are spherical-shaped but then elongate laterally to become crescent-like in shape. Subsequently, crescent-like mitochondria elongate continuously to coil tightly around the flagellum. Recently, disorganization of the mitochondrial sheath was reported in Glycerol kinase 2 (*Gk2*) disrupted mice. To analyze the disorganization of the mitochondrial sheath further, we generated *Gk2*-deficient mice using the CRISPR/Cas9 system and observed sperm mitochondria in testis using a freeze-fracture method with scanning electron microscopy. *Gk2*-disrupted spermatids show abnormal localization of crescent-like mitochondria, in spite of the initial proper alignment of spherical mitochondria around the flagellum, which causes abnormal mitochondrial sheath formation leading to exposure of the outer dense fibers. These results indicate that GK2 is essential for proper arrangement of crescent-like mitochondria to form the mitochondrial sheath during mouse spermatogenesis.

The sperm midpiece is characterized by the mitochondrial sheath that packs tightly around the axoneme and the nine outer dense fibers [1]. This mitochondrial sheath is formed by mitochondria that are recruited from the cytoplasm to the flagellum late in spermatogenesis [2]. In spermatogenesis, spherical-shaped mitochondria line up around the flagellum and elongate laterally to become crescent-like in shape. Subsequently, crescent-like mitochondria elongate continuously to coil tightly around the flagellum [3, 4]. Although formation of the mitochondrial sheath during spermatogenesis has been well described, the molecular mechanism of mitochondrial sheath formation remains unclear. To uncover the factors that are indispensable for the mitochondrial sheath formation, gene-manipulated animals have become indispensable. In fact, several mutant mice have been reported to exhibit morphological abnormalities in sperm mitochondria [5,6,7,8,9,10,11,12,13,14]. However, because the detailed behavior of mitochondria has not been observed, it is unclear how these factors are involved in the mitochondrial sheath formation.

The glycerol kinase family is composed of *Gk*, *Gk2*, *Gk5,* and *Gykl1* in mice. Glycerol kinase (GK) phosphorylates glycerol to glycerol 3-phosphate, which is an important step for the metabolism of glycerol that acts as the backbone of glyceride lipids [15]. *Gk* is expressed in various organs [16], and deficiency of GK causes an X-linked recessive disease in humans, which shows hyperglycerolemia associated with congenital adrenal hypoplasia and developmental delay [17, 18]. In addition, *Gk* KO mice show growth retardation, altered fat metabolism, autonomous glucocorticoid secretion, and neonatal death [19]. On the other hand, *Gk5*-deficienct mice show excessive amounts of cholesterol, triglycerides and ceramides in skin, and displayed hair loss that is caused by impaired hair growth and maintenance [20].

Both *Gykl1* and *Gk2* are thought to have arisen by the transposition of *Gk* located on the X chromosome [21, 22], and have high homology with *Gk* [21]. However, both *Gykl1* and *Gk2* show testis-specific expression, and the proteins have no glycerol kinase activity *in vitro* [21], unlike GK and GK5 [15, 20]. Recently, Chen *et al*., indicated that *Gykl1* or *Gk2* deficient mice show male infertility with disordered mitochondrial sheath formation [23]. In this study, we generated *Gk2*-deficient mice using the CRISPR/Cas9 system and observed the mitochondrial sheath formation using a freeze-fracture method with scanning electron microscopy (SEM) to further analyze its disorganization.

## Materials and Methods

### Experimental animals

All animal experiments were approved by the Animal Care and Use committee of the Research Institute for Microbial Diseases, Osaka University. Animals were housed in a temperature controlled environment with 12 h light cycles and free access to food and water. B6D2F1 (C57BL/6 × DBA2) mice or ICR mice were used as embryo donors or foster mothers, respectively. These animals were purchased from Japan SLC (Shizuoka, Japan). B6D2-Tg(CAG/Su9-DsRed2, Acr3-EGFP)RBGS002Osb (RBGS, Red Body Green Sperm) mice that were generated and maintained in our laboratory [24], were used for observing sperm mitochondria.

### Generation of Gk2-deficient mice

**Fig. 1.**
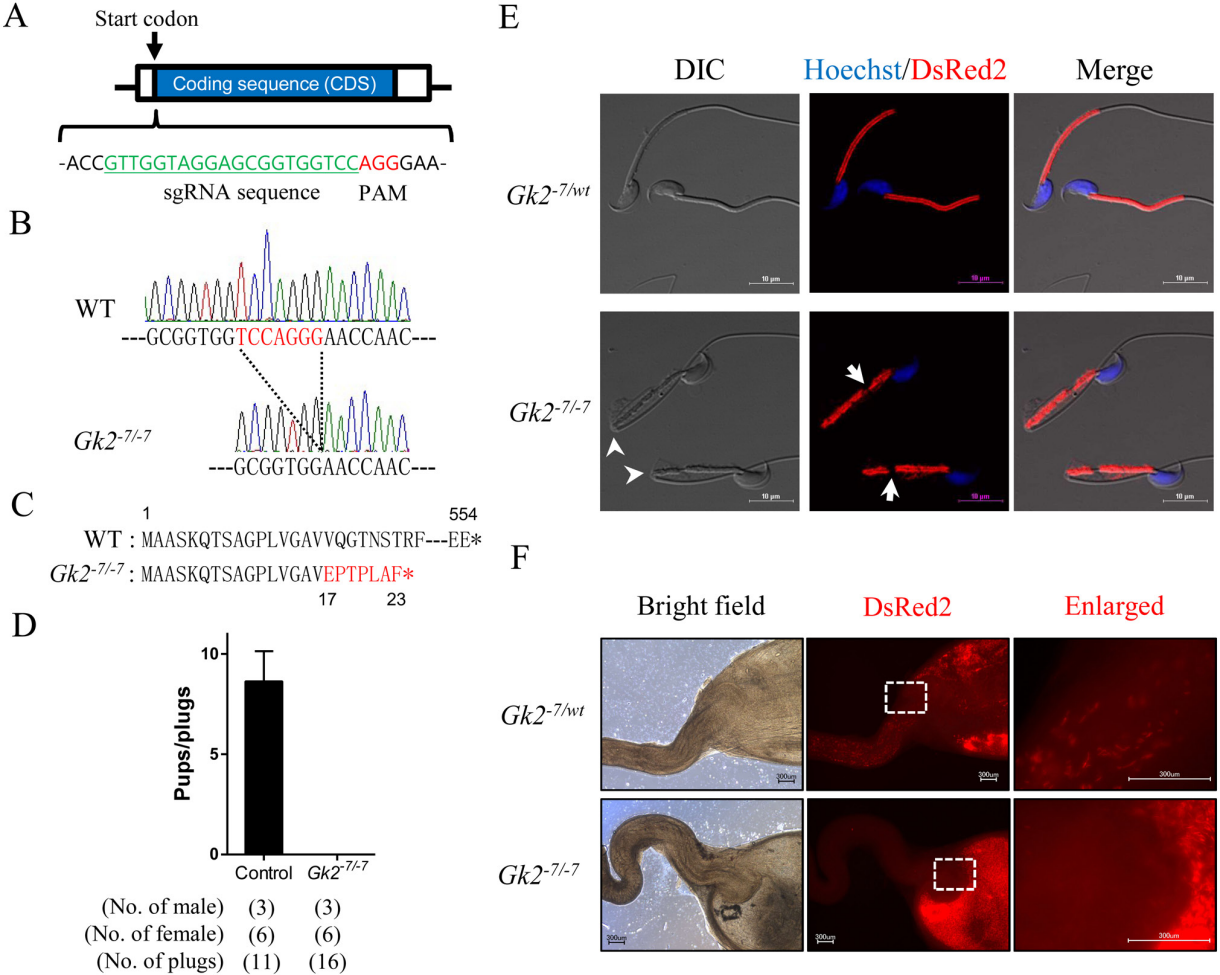
*Gk2* disrupted mice are male infertile because their spermatozoa cannot pass through the uterotubal junction (UTJ). (A) Design of sgRNA for generating *Gk2* KO mice. Guide sequence is highlighted in green. (B) Control and *Gk2^-7/-7^* alleles. Red letters indicate 7 bp deletion site. (C) Putative protein product of the *Gk2^-7/-7^* allele. Red letters indicates differing amino acids due to a frame shift. *, stop codon. (D) Average litter size of control and *Gk2* KO male mice. Error bars represent S.D. (E) Sperm morphology of control and *Gk2* KO mice in the RBGS background, which express mitochondria-targeted DsRed2 (red). Nuclei were stained with Hoechst 33342 (blue). Arrowheads indicate abnormal-bending. Arrows indicate fragmented mitochondrial sheath. Scale bars are 10 µm. (F) Imaging of spermatozoa inside the female reproductive tract 2−3 h after observing vaginal plugs. Left panels display reproductive organs under normal bright field conditions. Middle panels show red fluorescence of RBGS spermatozoa in the female reproductive tract. Right panels show enlarged images of the boxed areas. Scale bars are 300 µm.

To generate the *Gk2* KO mice, we prepared the pX330 plasmid (#42230, Addgene, Cambridge, MA, USA) expressing a chimeric sgRNA together with human codon-optimized Cas9 (hCas9) by ligating oligonucleotides into the *BbsI* site. Because *Gk2* is a single exon gene, we designed the sgRNAs that recognize sequences close to the start codon (Fig. 1A

). For avoiding off-target cleavage, we checked the specificity of sgRNA sequences with a homology search using Bowtie [25]. Plasmid DNA for injection was purified from bacterial colonies using a NucleoBond Xtra Midi kit (Macherey-Nagel, Düren, Germany), and Sanger sequenced using the primer (5'-TGGACTATCATATGCTTACC-3'). Before injection, we checked the DNA cleavage activity of the plasmid using the HEK 293T EGFP assay [26].

B6D2F1 female mice more than 8 weeks old were superovulated by injection of pregnant mare serum gonadotropin (PMSG, ASKA Animal Health, Tokyo, Japan) and human chorionic gonadotropin (hCG, ASKA Animal Health). After hCG injection, B6D2F1 females were mated with B6D2F1 males. Superovulated female mice with vaginal plugs were euthanized the next morning, and fertilized eggs were collected from the oviducts. The pronuclear stage eggs were injected with 5.0 ng/µl of pX330 plasmid targeting *Gk2*. The eggs were cultivated in KSOM medium overnight [27], and then transferred into the oviducts of pseudopregnant ICR females. Pups were obtained by C-section followed by fostering onto lactating mothers. Tail biopsies were performed for isolating genomic DNA for genotyping, and *Gk2* disrupted mice were maintained by sibling cross. *Gk2* KO female mice were mated with male B6D2-Tg(CAG/Su9-DsRed2, Acr3-EGFP)RBGS002Osb for generating *Gk2* KO mice expressing both EGFP in the acrosome and DsRed2 in the mitochondria (*Gk2* KO mice with RBGS).

### Genotype analysis

Polymerase chain reaction (PCR) was performed using KOD FX neo (TOYOBO, Osaka, Japan). The following primer sets were used for PCR: sense primer 5'-TGACTGGCTGTTGTGTCTCC-3' and antisense primer 5'- GTCGATGGTTCCAAACATGG-3'. PCR products were purified using a Wizard SV Gel and PCR Clean-Up System (Promega, Madison, WI, USA) kit, and Sanger sequenced with an ABI 3130*xl* Genetic Analyzer (Thermo Fisher Scientific, Waltham, MA, USA) using the antisense primer. All oligonucleotides were purchased from GeneDesign (Osaka, Japan).

### Fertility analysis of Gk2-disrupted mice

To confirm the fertility of *Gk2* KO male mice, natural mating tests were conducted. Three male mice were individually caged with two B6D2F1 females for 2 months. Both plug and pup numbers were checked around 1000 h every day to determine the number of copulations and litter size.

### Morphological and histological analysis of testis

*Gk2* KO male mice (11–12 weeks old) were euthanized and testes were dissected. After measuring the testicular weight, testes were fixed with Bouin’s fixative (Polysciences, Warrington, PA, USA). Fixed testes were embedded in paraffin, sectioned, rehydrated, and treated with 1% periodic acid for 10 min, followed by treatment with Sciff's reagent (Wako, Osaka, Japan) for 20 min. The sections were stained with Mayer’s haematoxylin solution prior to imaging, and observed using a BZ-X710 microscope (Keyence, Osaka, Japan).

### Morphological analysis of spermatozoa

Spermatozoa from *Gk2* KO male mice with RBGS were used for this analysis. Spermatozoa were collected from the cauda epididymis, and suspended in TYH medium [28] with 10 µg/ml of Hoechst 33342 (Thermo Fisher Scientific). After a 10 min incubation at 37°C under 5% CO_2_, a sperm suspension was mounted on MAS coated glass slides (Matsunami Glass, Osaka, Japan), and cover slipped (Matsunami Glass). Immunofluorescence and sperm shape were observed using a Nikon Eclipse Ti microscope connected to a Nikon C2 confocal module (Nikon, Tokyo, Japan).

The annulus of spermatozoa was stained with anti-human Septin4 (anti-SEPT4; Immuno-Biological Laboratories, Gunma, Japan). The spermatozoa mounted on glass slides with water-repellent fluororesin ink were dried followed by fixation with 4% paraformaldehyde (Wako) in phosphate-buffered saline (PBS), and blocked with 5% bovine serum albumin and 1% goat serum in PBS. The specimens were then incubated with anti-SEPT4 antibody at a dilution of 1/100 followed by staining with Alexa Fluor 488 (Thermo Fisher Scientific) at a dilution of 1/200. Immunofluorescence was analyzed with the Nikon confocal microscope set-up described above.

### Sperm motility analysis

Sperm motility analysis was performed as described previously [29]. Cauda epididymal spermatozoa were suspended and incubated in TYH medium. Sperm motility was then measured using the CEROS sperm analysis system (software version 12.3; Hamilton Thorne Biosciences, Beverly, MA, USA). Analysis settings were as described previously [30]. The motility of epididymal spermatozoa was recorded after 5 min or 2 h of incubation in TYH medium.

### In vitro fertilization

Spermatozoa were collected from the cauda epididymis of *Gk2* KO mice (11–12 weeks old) after euthanasia. Collected spermatozoa were capacitated *in vitro* for 2 h in TYH medium [28] at 37°C in a humidified atmosphere of 5% CO_2_ in air. B6D2F1 female mice more than 8 weeks old were superovulated, and the cumulus-intact eggs were collected from their oviduct 15–16 h after hCG injection, followed by incubation in TYH medium. To remove cumulus cells, eggs were treated with 1.0 mg/ml of hyaluronidase (Wako) for 5 min [31]. After removing cumulus cells, the zona pellucida was removed from eggs with 1.0 mg/ml collagenase (Sigma-Aldrich, St. Louis, MO, USA) [32]. Eggs were incubated with 2.0 × 10^5^ sperm/mL for 6 h. After 6 h co-incubation, formation of pronuclei were observed using a differential interference contrast (DIC) microscope (Olympus IX73, Tokyo Japan). Frequencies of 2-cell embryos were observed 24 h after insemination using the same DIC microscope. Fertilization rates of both cumulus-intact and -free eggs were examined by counting the number of 2-cell embryos, and that of zona-free eggs was examined by counting the number of eggs with pronuclei. The cumulus cell penetration assay was conducted as previously described [29].

### Imaging of sperm inside the female reproductive tract

Live imaging of spermatozoa inside the female reproductive tract was conducted as previously described [24]. B6D2F1 female mice more than 8 weeks old were superovulated and mated with *Gk2* KO male mice with RBGS 12–14 h after hCG injection. We checked for vaginal plugs every 30 min and sacrificed the female mice 2–3 h after observing a vaginal plug. Then we dissected female reproductive tracts and observed the spermatozoa inside the tract using a BZ-X710 microscope.

### Immunoblot analysis

Immunoblot analysis was conducted as previously described [33] with slight modifications. Testis or spermatozoa collected from both the cauda epididymis and vas deferens were obtained from sexually mature male mice. Proteins were extracted from testis or spermatozoa using a lysis buffer (TBS containing 1% Triton X-100 and 1/100 protease inhibitor cocktail). Proteins (20 µg per sample) were separated by SDS-PAGE under reducing conditions and transferred to polyvinylidene fluoride (PVDF) membrane using the Trans Blot Turbo system (BioRad, Munich, Germany). After blocking with 10% skim milk (Becton Dickinson, Cockeysville, MD, USA), the membrane was incubated with 1:2000 dilution of anti-ADAM3 antibody (clone 7C1.2; Merck, Darmstadt, Germany) overnight at 4°C, and then incubated with 1:5000 dilution of HRP-conjugated goat anti-mouse IgG (GE healthcare, Buckinghamshire, UK) at room temperature for 2 h. Chemiluminescence was detected by Chemi-Lumi One Super (Nacalai Tesque, Kyoto, Japan) using the Image Quant LAS 4000 mini (GE Healthcare).

### Morphological analysis of spermatozoa collected from the three regions of the epididymis

Epididymal spermatozoa (caput, corpus and cauda) were collected from *Gk2* KO male mice with RBGS. Collected spermatozoa from the caput or corpus epididymis were mounted on glass slides and suspended in PBS. Spermatozoa collected from the cauda epididymis were incubated for 30 min in TYH medium and mounted on glass slides. The sperm suspension was then coverslipped. Counting of spermatozoa was conducted using an Olympus BX50 DIC microscope, and more than 250 spermatozoa were counted for the frequencies of both bending sperm and fragmented mitochondrial sheath.

### Ultrastructural analysis using transmission electron microscopy (TEM)

Testes were dissected after perfusion fixation with 4% PFA in PBS under anesthesia, and immersed in 4% PFA for 6 h at 4°C. The organs were sliced into 2 mm × 2 mm × 2 mm pieces with safety razors, immersed in 1% glutaraldehyde in 30 mM HEPES (pH 7.8) overnight at 4°C, and washed three times (5 min each) in 30 mM HEPES. Tissues were postfixed in 1% OsO_4_ and 0.5% potassium ferrocyanide in 30 mM HEPES for 1 h at room temperature. After being washed with distilled water, samples were dehydrated with a graded series of ethanol solutions (50, 70, 90%) on ice, and in 100% ethanol for 10 min at room temperature. Dehydrated samples were incubated twice for 5 min in 100% propylene oxide (PO), and then placed in a mixture of PO and epoxy resin for 1 h at room temperature. Sample tissues were incubated in a pure epoxy resin mixture twice for 1 h at room temperature, and embedded in epoxy resin for 2 days at 60°C. Eighty nm ultrathin sections were cut and stained with 2% uranyl acetate solution for 30 min, briefly washed three times with distilled water, stained with a lead staining solution for 2 min, and washed three times with distilled water. The sample were examined using a JEM-1400 Plus electron microscope (JEOL, Tokyo, Japan) at 80 kV with a CCD Veleta 2K × 2K camera (Olympus). Stages of the epithelial cycle were identified based on the morphological characteristics of the spermatids, in particular their nucleus and acrosomic system [2].

### Ultrastructural analysis of sperm mitochondria in spermatogenesis using SEM

Tissue samples were prepared with the freeze-fracture method as described previously [3] with slight modifications. The testes were dissected from the mice and sliced into rings 5 mm thick using safety razors. The specimens were fixed in 1% OsO_4_ in 0.1 M phosphate buffer (pH 7.4) for 1 h at 4°C, immersed in 50% dimethyl sulfoxide (DMSO) solution, and cracked with a freeze-cracking apparatus TF-2 (Eiko, Tokyo, Japan). After the specimens were rinsed, they were transferred to buffered 0.1% OsO_4_ and left standing for 48–72 h at 20°C. The samples were then postfixed with buffered 1% OsO_4_ for 1 h at 4°C, and stained with 2% tannic acid solution (Wako) for 2 h followed by buffered 1% OsO_4_ for 1 h. The specimens were dehydrated in a graded series of ethanol solutions (50, 70, 90, 100, 100, 100%), and critical point dried using a Samdri-PVT-3D system (Tousimis, Rockland, MD, USA). The specimens were then mounted on sample stages, and coated with osmium using an osmium coater HPC-30W (Vacuum Device, Ibaraki, Japan). The samples were observed with an S-4800 field emission scanning electron microscope (Hitachi, Tokyo, Japan).

### Statistical analysis

Statistical analysis were performed using a two-tailed student's *t*-test (* P < 0.05, ** P < 0.01) by GraphPad Prism 6 (GraphPad, San Diego, CA, USA). Data represent the means ± standard deviation (SD).

### Data availability

The *Gk2* KO mouse strain used in this study was deposited under the name B6D2-*Gk2^em1Osb^*, and available through either the Riken BioResource Center (Riken BRC; Tsukuba, Japan) or the Center for Animal Resources and Development, Kumamoto University (CARD; Kumamoto, Japan). The stock ID number of *Gk2* KO mouse strain is 09856 (Riken BRC) or 2476 (CARD), respectively. All other data are available from the authors upon reasonable request.

## Results

### Generation of Gk2 disrupted mice

To study how GK2 is involved in mitochondrial sheath formation, we generated *Gk2* KO mice using the CRISPR/Cas9 system. The cleavage target site was designed just after the start codon of *Gk2* (Fig. 1A), and pX330 plasmid that expressed a sgRNA and human CAS9 protein was constructed. DNA cleavage activity of this plasmid was checked by the HEK 293T EGFP assay [26]. *Gk2* disrupted mice were generated by microinjecting 5.0 ng/µL of pX330 plasmid into oocytes. Of the 135 fertilized oocytes that had been injected, 71 eggs were transplanted into oviducts of pseudopregnant females. A total of 22 founder mice (F0) were born, and 5 pups possessed a mutation. A mutant line that had a 7-bp deletion was used for this study (Fig. 1B). The 7-bp deletion caused a frameshift mutation affecting the 17th amino acid, and leading to a premature stop at position 23 (Fig. 1C). *Gk2^-7/-7^* mice were viable and showed no overt abnormalities. For analyzing the off-target effect of *Gk2*-targeting sgRNA, we checked the DNA sequence of both *Gk* and *Gykl1* in *Gk2* KO mice, and confirmed no mutations in their coding sequence.

### Gk2 KO spermatozoa cannot transit the uterotubal junction (UTJ) due to reduced motility, which induces male infertility

To ensure that the phenotype of *Gk2^-7/-7^* mice are the same as that of previous report [23], we checked their fertility. *Gk2^-7/-7^* male mice were mated with wild type (WT) females for 2 months. Although vaginal plugs were observed 16 times, no pups were born from *Gk2^-7/-7^* male mice (Fig. 1D). In contrast, *Gk2^-7/-7^* female mice were fertile with the average litter size being 7.5 ± 1.3 (mean ± S.D., *n* = 12). To see the effect of *Gk2* disruption on testis, both morphological and histological analysis were conducted. However, there were no significant differences in gross appearance (Supplementary Fig. 1A: online only), organ weight (Supplementary Fig. 1B), and histological analysis (Supplementary Fig. 1C) of testis.

To confirm the sperm abnormality reported previously [23], we observed the spermatozoa obtained from our *Gk2* KO mice. An abnormal bending of the tail and fragmented mitochondrial sheath were observed in *Gk2* KO spermatozoa collected from the cauda epididymis (Fig. 1E), which are consistent with the previous report [23]. Because spermatozoa were bent near the annulus (posterior end of the midpiece), we observed SEPT4 localization, which is essential for establishment of the annulus [7]. Although almost every KO spermatozoa were bent near the annulus, SEPT4 localization was normal (Supplementary Fig. 2A: online only).

To reveal the cause of infertility, we analyzed sperm motility using computer-assisted spermatozoa analysis (CASA). The percentage of motile sperm in *Gk2* KO mice was lower than that of control both before and after induction of capacitation (Supplementary Fig. 2B). In addition, *Gk2* KO spermatozoa showed significantly lower values in average path velocity (VAP), straight line velocity (VSL), and curvilinear velocity (VCL) at both 5 and 120 min of incubation (Supplementary Fig. 2C). To check the fertility of *Gk2* KO spermatozoa further *in vitro*, we conducted *in vitro* fertilization (IVF), and found that the fertilization rate of *Gk2^-7/-7^* spermatozoa with both cumulus-intact and cumulus-free oocyte were reduced (Supplementary Fig. 2D). However, the fertilization rate of *Gk2-7/-7* spermatozoa with zona-free oocytes was comparable to that of control (Supplementary Fig. 2D). To check the behavior of *Gk2* KO spermatozoa within the cumulus layer, the cumulus cell penetration assay was conducted [29]. Based on the results, *Gk2* KO spermatozoa could pass through the cumulus cell layers (Supplementary Fig. 2E). These findings suggest that *Gk2* KO spermatozoa can undergo the acrosome reaction and fuse with eggs, but penetration of the zona pellucida is difficult for *Gk2* KO spermatozoa.

We then observed sperm migration into the oviduct using *Gk2* KO male mice with RBGS that expresses EGFP in the acrosome and DsRed2 in the mitochondria. Although control spermatozoa can transit into oviduct through the uterotubal junction (UTJ), no *Gk2^-7/-7^* spermatozoa can pass through the UTJ (Fig. 1F). Because processing of ADAM3 in the epididymis is essential for passing through the UTJ [33,34,35], we examined the processing of ADAM3 by western blot analysis. In *Gk2^-7/-7^* mice, the unprocessed form of ADAM3 in testicular spermatozoa and the processed form in epididymal spermatozoa were detected normally (Supplementary Fig. 2F). These results indicate that *Gk2^-7/-7^* KO mice show male infertility due to an impairment of sperm passage through the UTJ despite proper ADAM3 processing. Because sperm motility is also important for transiting through the UTJ [29, 36, 37], the decreased sperm motility due to morphological abnormality of spermatozoa can be the cause of infertility of *Gk2* KO mice.

### Fragmentation of the mitochondrial sheath is observed earlier than tail bending

**Fig. 2.**
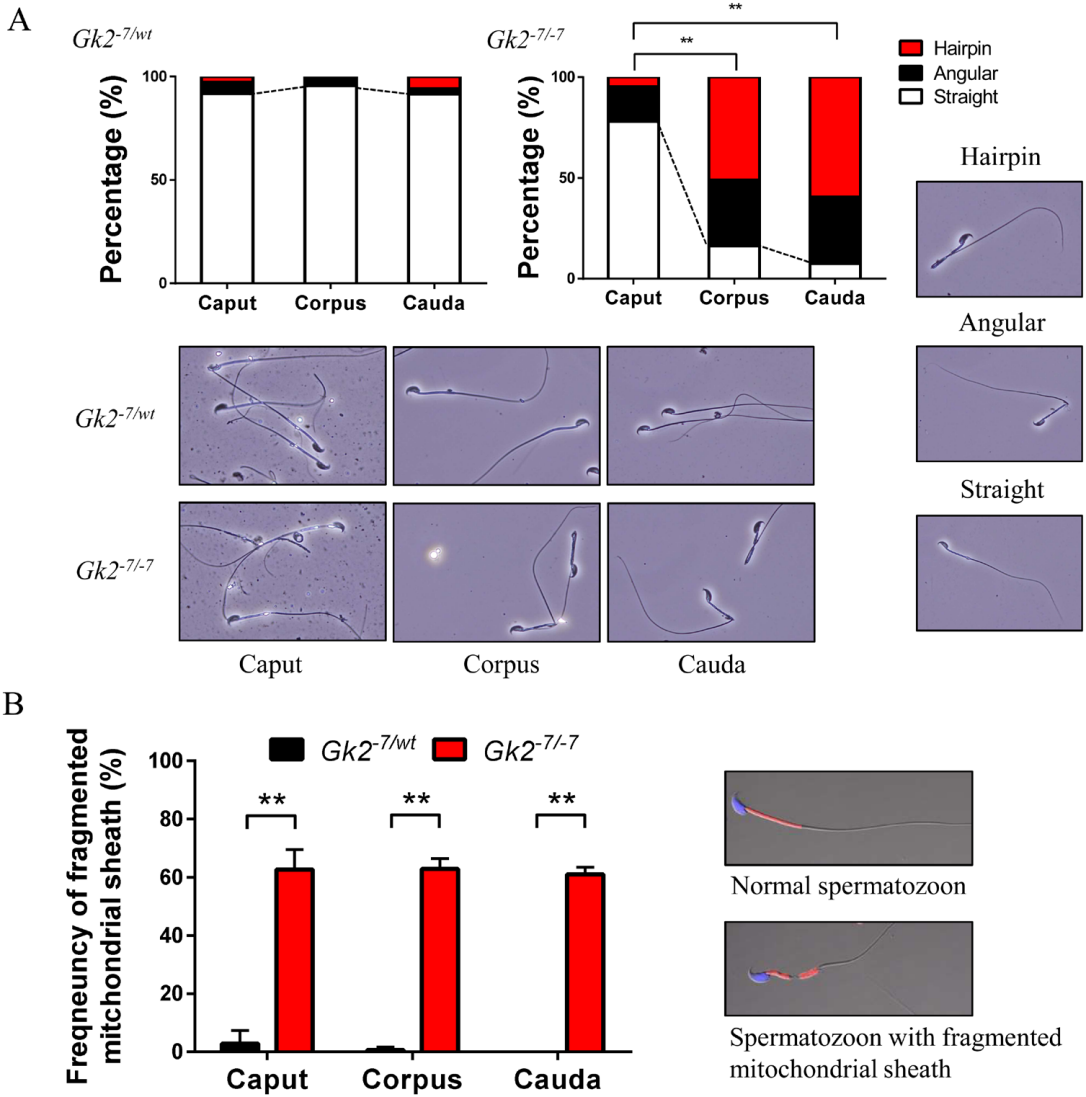
Mitochondrial disorganization of *Gk2* disrupted spermatozoa appears before sperm bending. (A) Graphs indicate frequencies of bending spermatozoa collected from the three sections of epididymis. Lower panels show representative images of spermatozoa collected from the three sections of epididymis. Spermatozoon shape is categorized as Hairpin, Angular, or Straight (displayed in right panels). ** P < 0.01, Student’s *t *test. (B) Graph indicates the frequencies of fragmented mitochondrial sheath collected from the three sections of epididymis. Right panels are representative images of spermatozoon with or without a fragmented mitochondrial sheath. ** P < 0.01, Student’s *t* test, Error bars represent S.D.

To analyze when the morphological abnormalities in *Gk2^-7/-7^* spermatozoa emerge, spermatozoa were collected from three sections of the epididymis (caput, corpus and cauda) to monitor the frequencies of both bending spermatozoa and fragmented sperm mitochondria. Although bending spermatozoa were observed in both corpus and cauda epididymis, few bending spermatozoa were observed in caput epididymis (Fig. 2A

). This result indicates that the phenotype of bending spermatozoa becomes worse during sperm maturation in the epididymis. In contrast, frequencies of fragmented mitochondrial sheath were high in *Gk2^-7/-7^* spermatozoa in the caput epididymis and stayed constant (Fig. 2B). This result suggests that mitochondrial disorganization in *Gk2^-7/-7^* spermatozoa occurs before sperm bending.

### Gk2 KO spermatids exhibit abnormal arrangement of crescent-like mitochondria, which causes a disorganization of the mitochondrial sheath

**Fig. 3.**
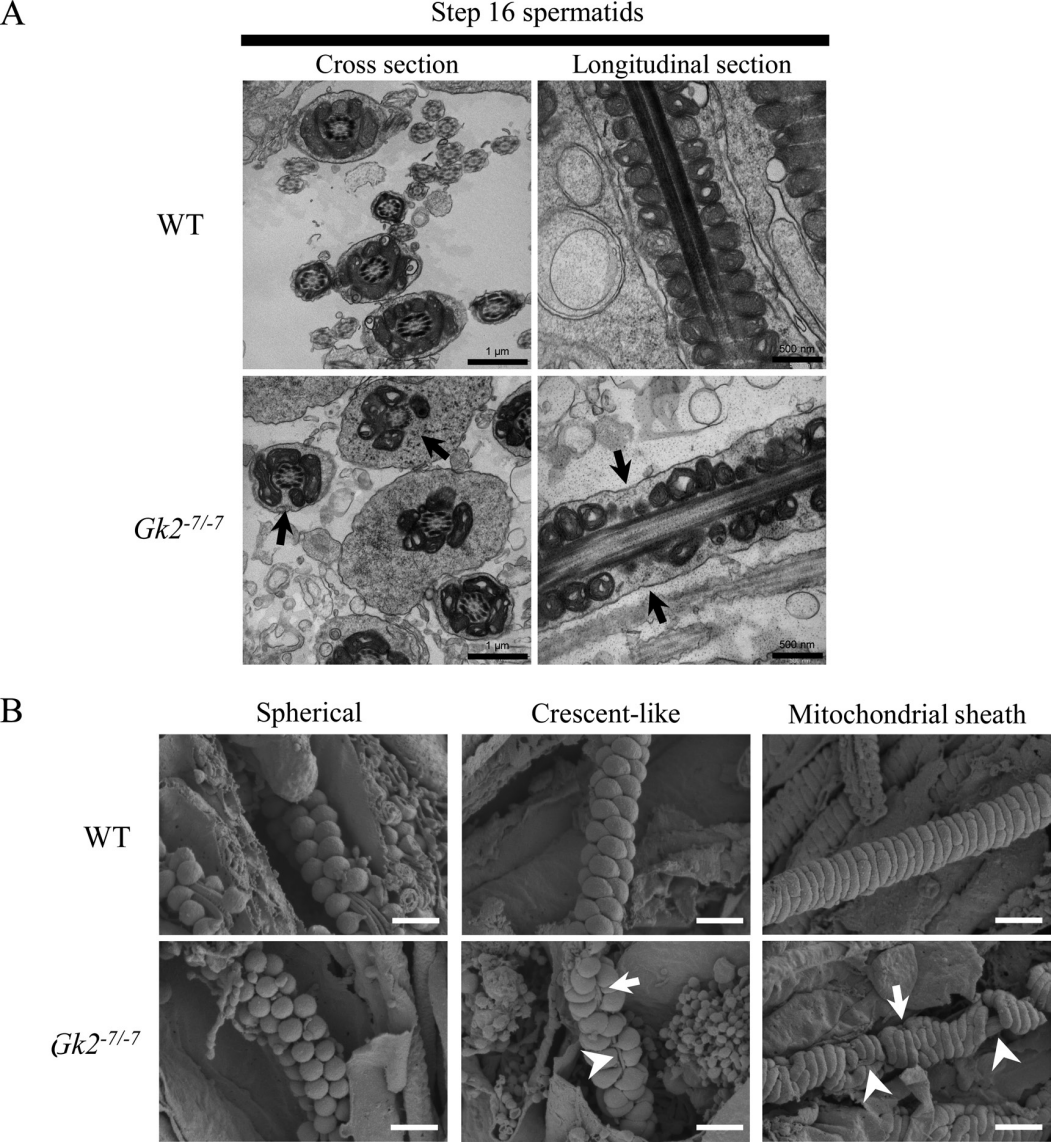
*Gk2* deficient spermatids show aberrant mitochondrial sheath formation caused by misalignment of crescent-like mitochondria. (A) Ultrastructural images of step 16 spermatids (stage VII) analyzed by transmission electron microscopy (TEM). Arrows indicate the midpiece not surrounded by mitochondria. Scale bars of cross section and longitudinal section are 1 µm and 500 nm, respectively. (B) Mitochondrial sheath development during spermatogenesis observed by scanning electron microscopy (SEM). During spermatogenesis, spherical mitochondria align around flagellum (left), and change their shape to crescent-like mitochondria to surround the flagellum (middle), and then the mitochondria continue to elongate to form mitochondrial sheath (right). Arrows show breaks of aligned mitochondria. Arrowheads show exposed outer dense fiber. Scale bars are 1 µm.

Because mitochondrial sheath fragmentation in *Gk2^-7/-7^* spermatozoa were observed in the caput epididymis, we observed spermatids in the testis. First, we observed spermatids using transmission electron microscope (TEM). As expected, bending near the annulus was not observed in step 16 spermatids in *Gk2^-7/-7^* testis (Supplementary Fig. 3A: online only). Although no morphological aberrations were observed in step 15 spermatids, step 16 spermatids contained midpieces not surrounded by mitochondria where coiled mitochondria are typically observed (Fig. 3A

). In contrast, the axonemes of *Gk2^-7/-7^* spermatozoa were normal. Occasionally we observed vacuole-like mitochondria in step16 spermatids, but these were also observed in WT, suggesting these vacuoles may normally be present.

To further analyze the mitochondrial behavior, we observed mitochondrial sheath development using the freeze-fracture method with SEM [3]. We observed the formation of the mitochondrial sheath with proper crescent-like mitochondrial alignment in WT spermatids (Fig. 3B, upper middle). In contrast, despite the normal alignment of spherical-shaped mitochondria (Fig. 3B, lower left), alignment of crescent-like mitochondria was disorganized in *Gk2*-deficient spermatids (Fig. 3B, lower middle). Subsequently, an aberrant mitochondrial sheath was formed (Fig. 3B, lower right). Crescent-like mitochondria should align around the flagellum in a zig-zag manner [3, 4], but *Gk2^-7/-7^* sperm mitochondria were randomly aligned, which sometimes leads to breaks of aligned mitochondria or exposure of outer dense fibers (Fig. 3B, lower middle). In addition, the same mitochondrial abnormality is still observed even after mitochondrial coiling (Fig.3B, lower right). However, no changes were found in the structure of mitochondrial cristae. Taken together, *Gk2^-7/-7^* spermatids exhibit abnormal arrangement of crescent-like mitochondria, which causes disorganization of the mitochondrial sheath.

## Discussion

The molecular mechanism of sperm mitochondrial sheath formation has been largely unknown. Because morphological defects in mitochondria are one cause of human asthenozoospermia [38, 39], revealing the mechanism is beneficial for developing new fertility treatments and male contraceptives. Recently, Chen *et al*., revealed that GK2 is specifically localized to the mitochondria in spermatids, and *Gk2* KO male mice are infertile. In addition, they described that *Gk2* KO spermatozoa shows disordered mitochondrial sheath formation such as fragmentation of mitochondria and short mitochondrial sheath [23]. However, how GK2 is involved in mitochondrial sheath formation remains unknown. Here, we studied the mechanism of disordered mitochondrial sheath formation in *Gk2* KO spermatozoa using the freeze-fracture method with SEM. As a result, we revealed that GK2 is essential for proper arrangement of crescent-like mitochondria to form the mitochondrial sheath during mouse spermatogenesis (Fig. 3B and Supplementary Fig. 3B). In addition, we demonstrated that *Gk2*-disrupted spermatozoa cannot pass through the UTJ due to reduced sperm motility caused by morphological abnormalities (Fig. 1F, Supplementary Figs. 2B and 2C). We propose that this is the main reason of infertility of *Gk2* KO male mice.

In the present study, we generated *Gk2* KO mice using the CRISPR/Cas9 system independently of the previous report [23], and the phenotypes observed in *Gk2^-7/-7^* spermatozoa such as bending of the tail and mitochondrial fragmentation were the same as those of the previous report [23]. Both abnormal tail bending and mitochondrial fragmentation were observed in the cauda epididymal spermatozoa of *Gk2^-7/-7^* mice (Fig. 1E). However, because tail bending was not detected in *Gk2^-7/-7^* spermatids in testis (Fig. 3A and Supplementary Fig. 3A), the bending of the tail could have arisen as a secondary effect of mitochondrial sheath disorganization. Therefore, although mutant mice of *Sepp1*, *Sept4*, *mGpx4*, and *Ddhd1* (*PA-PLA_1_*) were reported to have a mitochondrial abnormality with tail bending [6, 7, 9, 10, 13], the bending might be a secondary effect derived from mitochondrial disorganization as in *Gk2*-disrupted mice.

As a result of ultrastructural examination using the freeze-fracture method, both misaligned crescent-like mitochondria and exposure of outer dense fibers were observed in *Gk2^-7/-7^* spermatids (Fig. 3B and Supplementary Fig. 3B). Because each mitochondrion first localizes to the flagellum and elongates laterally from two directions to coil around the flagellum [4], exposure of outer dense fibers observed in testis (Fig. 3B and Supplementary Fig. 3B) is thought to be induced by the misalignment of crescent-like mitochondria. In addition, it is assumed that the mitochondrial fragmentation observed in cauda epididymal spermatozoa (Fig. 1E and 2B) is caused by the exposure of outer dense fibers observed in testis (Fig. 3B and Supplementary Fig. 3B). Although this conclusion is readily apparent from images obtained with SEM, it is difficult to discern with TEM analysis (Fig. 3A). Specifically, abnormal arrangement of both spherical and crescent-like mitochondrial are difficult to detect by TEM analysis. Therefore, the freeze-fracture method with SEM [3] can be a powerful tool for understanding the mitochondrial sheath formation, and other aspects of sperm morphology.

Although a member of the glycerol kinase family, GK2 lacks glycerol kinase activity *in vitro* [21]. Therefore, it is likely that GK2 may have other functions independent of glycerol kinase activity. *Chen et al*., revealed that both GYKL1 and GK2 interact with PLD6 (MitoPLD), and induce phosphatidic acid (PA)-dependent mitochondrial clustering *in vitro* [23]. How these proteins regulate sperm-mitochondrial dynamics is unknown, but PA that are regulated by GK2 may be involved in the proper arrangement of crescent-like mitochondria. This idea is supported by the result that mitochondria in cells that overexpresses mitoPLD aggregate into a compound structure [40]. Namely, mitochondrial aggregation induced by PA might be important for proper arrangement of crescent-like mitochondria. Although further studies are needed to understand how GK2 is involved in the arrangement of crescent-like mitochondria, GK2 is important factor for proper arrangement of crescent-like mitochondria to form the mitochondrial sheath during mouse spermatogenesis.

## Supplementary

Supplement Figures

## References

[1] Fawcett DW The mammalian spermatozoon. Dev Biol 1975; 44: 394–436. 805734 10.1016/0012-1606(75)90411-x

[2] Russell L Ettlin R Sinha Hikim A Clegg E Histological and histopathological evaluation of the testis. 1990. Cache River Press.

[3] Otani H Tanaka O Kasai K Yoshioka T Development of mitochondrial helical sheath in the middle piece of the mouse spermatid tail: regular dispositions and synchronized changes. Anat Rec 1988; 222: 26–33. 3189885 10.1002/ar.1092220106

[4] Ho H-C Wey S Three dimensional rendering of the mitochondrial sheath morphogenesis during mouse spermiogenesis. Microsc Res Tech 2007; 70: 719–723. 17457821 10.1002/jemt.20457

[5] Bouchard MJ Dong Y McDermott BM Jr Lam DH Brown KR Shelanski M Bellvé AR Racaniello VR Defects in nuclear and cytoskeletal morphology and mitochondrial localization in spermatozoa of mice lacking nectin-2, a component of cell-cell adherens junctions. Mol Cell Biol 2000; 20: 2865–2873. 10733589 10.1128/mcb.20.8.2865-2873.2000 PMC85510

[6] Olson GE Winfrey VP Nagdas SK Hill KE Burk RF Selenoprotein P is required for mouse sperm development. Biol Reprod 2005; 73: 201–211. 15744015 10.1095/biolreprod.105.040360

[7] Kissel H Georgescu M-M Larisch S Manova K Hunnicutt GR Steller H The Sept4 septin locus is required for sperm terminal differentiation in mice. Dev Cell 2005; 8: 353–364. 15737931 10.1016/j.devcel.2005.01.021

[8] Suzuki-Toyota F Ito C Toyama Y Maekawa M Yao R Noda T Iida H Toshimori K Factors maintaining normal sperm tail structure during epididymal maturation studied in Gopc-/- mice. Biol Reprod 2007; 77: 71–82. 17360959 10.1095/biolreprod.106.058735

[9] Schneider M Förster H Boersma A Seiler A Wehnes H Sinowatz F Neumüller C Deutsch MJ Walch A Hrabé de Angelis M Wurst W Ursini F Roveri A Maleszewski M Maiorino M Conrad M Mitochondrial glutathione peroxidase 4 disruption causes male infertility. FASEB J 2009; 23: 3233–3242. 19417079 10.1096/fj.09-132795

[10] Imai H Hakkaku N Iwamoto R Suzuki J Suzuki T Tajima Y Konishi K Minami S Ichinose S Ishizaka K Shioda S Arata S Nishimura M Naito S Nakagawa Y Depletion of selenoprotein GPx4 in spermatocytes causes male infertility in mice. J Biol Chem 2009; 284: 32522–32532. 19783653 10.1074/jbc.M109.016139 PMC2781666

[11] Johnson AR Craciunescu CN Guo Z Teng Y-W Thresher RJ Blusztajn JK Zeisel SH Deletion of murine choline dehydrogenase results in diminished sperm motility. FASEB J 2010; 24: 2752–2761. 20371614 10.1096/fj.09-153718 PMC2909292

[12] Colley SM Wintle L Searles R Russell V Firman RC Smith S Deboer K Merriner DJ Genevieve B Bentel JM Stuckey BGA Phillips MR Simmons LW de Kretser DM O’Bryan MK Leedman PJ Loss of the nuclear receptor corepressor SLIRP compromises male fertility. PLoS One 2013; 8: e70700. 23976951 10.1371/journal.pone.0070700 PMC3744554

[13] Baba T Kashiwagi Y Arimitsu N Kogure T Edo A Maruyama T Nakao K Nakanishi H Kinoshita M Frohman MA Yamamoto A Tani K Phosphatidic acid (PA)-preferring phospholipase A1 regulates mitochondrial dynamics. J Biol Chem 2014; 289: 11497–11511. 24599962 10.1074/jbc.M113.531921 PMC4036285

[14] Mi Y Shi Z Li J Spata19 is critical for sperm mitochondrial function and male fertility. Mol Reprod Dev 2015; 82: 907–913. 26265198 10.1002/mrd.22536

[15] Lin EC Glycerol utilization and its regulation in mammals. Annu Rev Biochem 1977; 46: 765–795. 197882 10.1146/annurev.bi.46.070177.004001

[16] Ohira RH Dipple KM Zhang Y-H McCabe ERB Human and murine glycerol kinase: influence of exon 18 alternative splicing on function. Biochem Biophys Res Commun 2005; 331: 239–246. 15845384 10.1016/j.bbrc.2005.03.143

[17] Guggenheim MA McCabe ERB Roig M Goodman SI Lum GM Bullen WW Ringel SP Glycerol kinase deficiency with neuromuscular, skeletal, and adrenal abnormalities. Ann Neurol 1980; 7: 441–449. 6249182 10.1002/ana.410070509

[18] Lewis B Harbord M Keenan R Carey W Harrison R Robertson E Isolated glycerol kinase deficiency in a neonate. J Child Neurol 1994; 9: 70–73. 7512107 10.1177/088307389400900118

[19] Huq AH Lovell RS Ou C-N Beaudet AL Craigen WJ X-linked glycerol kinase deficiency in the mouse leads to growth retardation, altered fat metabolism, autonomous glucocorticoid secretion and neonatal death. Hum Mol Genet 1997; 6: 1803–1809. 9302256 10.1093/hmg/6.11.1803

[20] Zhang D Tomisato W Su L Sun L Choi JH Zhang Z Wang KW Zhan X Choi M Li X Tang M Castro-Perez JM Hildebrand S Murray AR Moresco EMY Beutler B Skin-specific regulation of SREBP processing and lipid biosynthesis by glycerol kinase 5. Proc Natl Acad Sci USA 2017; 114: E5197–E5206. 28607088 10.1073/pnas.1705312114 PMC5495269

[21] Pan Y Decker WK Huq AHHM Craigen WJ Retrotransposition of glycerol kinase-related genes from the X chromosome to autosomes: functional and evolutionary aspects. Genomics 1999; 59: 282–290. 10444329 10.1006/geno.1999.5874

[22] Sargent CA Affara NA Bentley E Pelmear A Bailey DMD Davey P Dow D Leversha M Aplin H Besley GTN Ferguson-Smith MA Cloning of the X-linked glycerol kinase deficiency gene and its identification by sequence comparison to the Bacillus subtilis homologue. Hum Mol Genet 1993; 2: 97–106. 8499912 10.1093/hmg/2.2.97

[23] Chen Y Liang P Huang Y Li M Zhang X Ding C Feng J Zhang Z Zhang X Gao Y Zhang Q Cao S Zheng H Liu D Songyang Z Huang J Glycerol kinase-like proteins cooperate with Pld6 in regulating sperm mitochondrial sheath formation and male fertility. Cell Discov 2017; 3: 17030. 28852571 10.1038/celldisc.2017.30 PMC5566117

[24] Hasuwa H Muro Y Ikawa M Kato N Tsujimoto Y Okabe M Transgenic mouse sperm that have green acrosome and red mitochondria allow visualization of sperm and their acrosome reaction in vivo. Exp Anim 2010; 59: 105–107. 20224175 10.1538/expanim.59.105

[25] Langmead B Trapnell C Pop M Salzberg SL Ultrafast and memory-efficient alignment of short DNA sequences to the human genome. Genome Biol 2009; 10: R25. 19261174 10.1186/gb-2009-10-3-r25 PMC2690996

[26] Mashiko D Fujihara Y Satouh Y Miyata H Isotani A Ikawa M Generation of mutant mice by pronuclear injection of circular plasmid expressing Cas9 and single guided RNA. Sci Rep 2013; 3: 3355. 24284873 10.1038/srep03355 PMC3842082

[27] Ho Y Wigglesworth K Eppig JJ Schultz RM Preimplantation development of mouse embryos in KSOM: augmentation by amino acids and analysis of gene expression. Mol Reprod Dev 1995; 41: 232–238. 7654376 10.1002/mrd.1080410214

[28] Toyoda Y Yokoyama M The Early History of the TYH Medium for in vitro Fertilization of Mouse Ova. J Mamm Ova Res 2016; 33: 3–10.

[29] Miyata H Satouh Y Mashiko D Muto M Nozawa K Shiba K Fujihara Y Isotani A Inaba K Ikawa M Sperm calcineurin inhibition prevents mouse fertility with implications for male contraceptive. Science 2015; 350: 442–445. 26429887 10.1126/science.aad0836

[30] Goodson SG Zhang Z Tsuruta JK Wang W O’Brien DA Classification of mouse sperm motility patterns using an automated multiclass support vector machines model. Biol Reprod 2011; 84: 1207–1215. 21349820 10.1095/biolreprod.110.088989 PMC3099585

[31] Tokuhiro K Ikawa M Benham AM Okabe M Protein disulfide isomerase homolog PDILT is required for quality control of sperm membrane protein ADAM3 and male fertility [corrected]. Proc Natl Acad Sci USA 2012; 109: 3850–3855. 22357757 10.1073/pnas.1117963109 PMC3309714

[32] Inoue N Ikawa M Isotani A Okabe M The immunoglobulin superfamily protein Izumo is required for sperm to fuse with eggs. Nature 2005; 434: 234–238. 15759005 10.1038/nature03362

[33] Yamaguchi R Yamagata K Ikawa M Moss SB Okabe M Aberrant distribution of ADAM3 in sperm from both angiotensin-converting enzyme (Ace)- and calmegin (Clgn)-deficient mice. Biol Reprod 2006; 75: 760–766. 16870943 10.1095/biolreprod.106.052977

[34] Ikawa M Inoue N Benham AM Okabe M Fertilization: a sperm’s journey to and interaction with the oocyte. J Clin Invest 2010; 120: 984–994. 20364096 10.1172/JCI41585 PMC2846064

[35] Okabe M The cell biology of mammalian fertilization. Development 2013; 140: 4471–4479. 24194470 10.1242/dev.090613

[36] Gaddum-Rosse P Some observations on sperm transport through the uterotubal junction of the rat. Am J Anat 1981; 160: 333–341. 6894349 10.1002/aja.1001600309

[37] Fujihara Y Miyata H Ikawa M Factors controlling sperm migration through the oviduct revealed by gene-modified mouse models. Exp Anim 2018; 67: 91–104. 29353867 10.1538/expanim.17-0153 PMC5955741

[38] Gopalkrishnan K Padwal V D’Souza R Severe asthenozoospermia: a structural and functional study. Int J Androl 1995; 18: 67–74. 10.1111/j.1365-2605.1995.tb00642.x 7558392

[39] Pelliccione F Micillo A Cordeschi G D’Angeli A Necozione S Gandini L Lenzi A Francavilla F Francavilla S Altered ultrastructure of mitochondrial membranes is strongly associated with unexplained asthenozoospermia. Fertil Steril 2011; 95: 641–646. 20840880 10.1016/j.fertnstert.2010.07.1086

[40] Choi SY Huang P Jenkins GM Chan DC Schiller J Frohman MA A common lipid links Mfn-mediated mitochondrial fusion and SNARE-regulated exocytosis. Nat Cell Biol 2006; 8: 1255–1262. 17028579 10.1038/ncb1487

