# Supplementary material for: Glycerol kinase 2 is essential for proper arrangement of crescent-like mitochondria to form the mitochondrial sheath during mouse spermatogenesis
Source: J Reprod Dev. 2019 Jan 21;65(2):155–62. doi: 10.1262/jrd.2018-136 (PMC6473107; doi:10.1262/jrd.2018-136)
Supplement: Supplement Figures [file jrd-65-155-s001.pdf]

Figure. S1 (Shimada *et al.*)

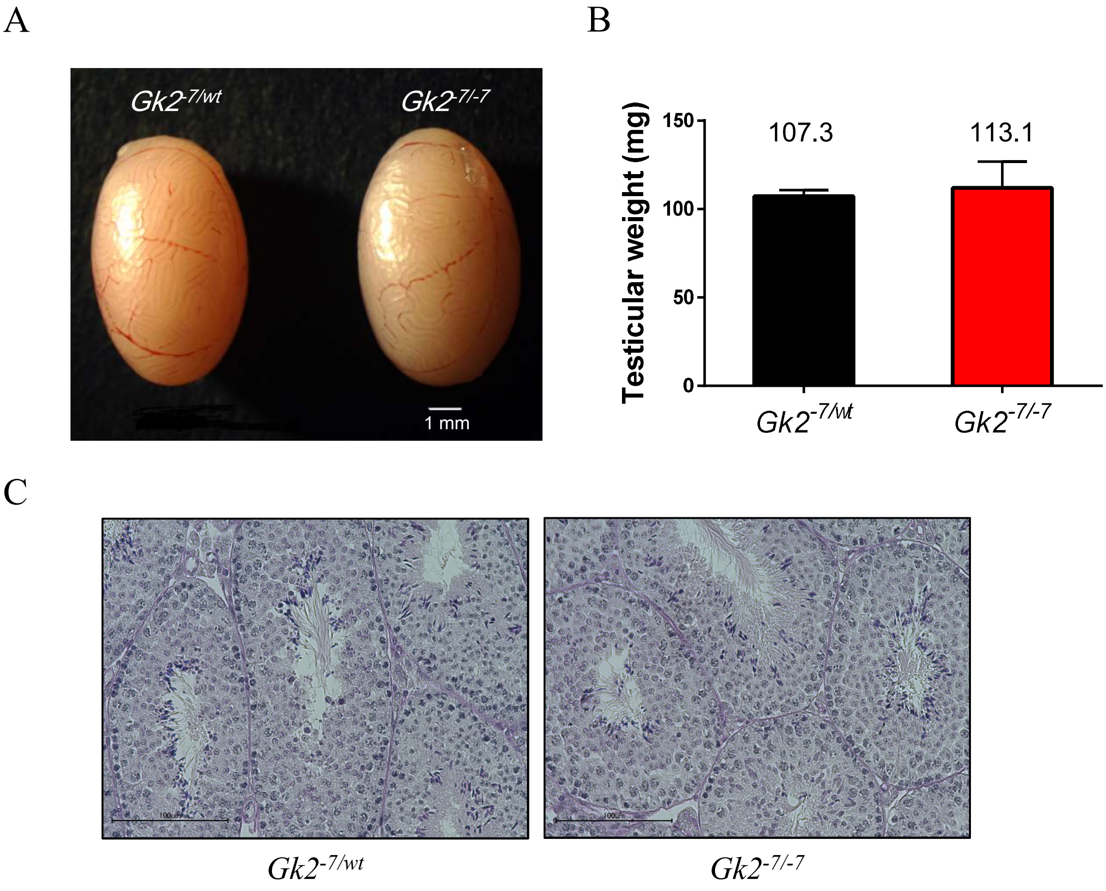

Fig. S1. No obvious changes were found in *Gk2*-difficient mouse testis

(A) Gross morphology of control and *Gk2* KO testis. Scale bar is 1mm.

(B) Average weight of control and *Gk2* KO testis. Error bars represent S.D.

(C) PAS staining of testis sampled from control and *Gk2* KO male mice. Scale bars are 100 µm.

Figure. S2 (Shimada *et al.*)

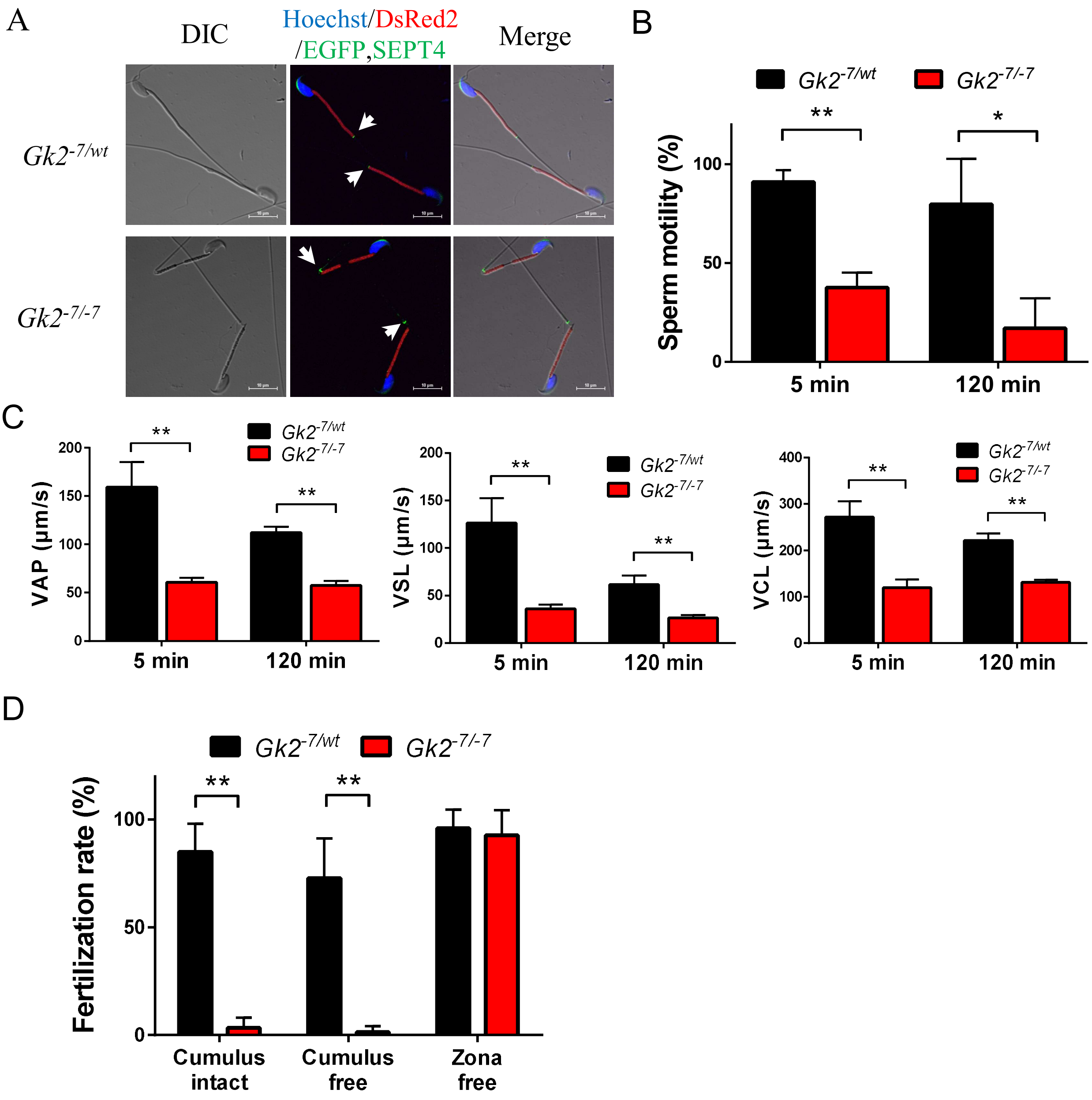

Figure. S2 (Shimada *et al.*)

E

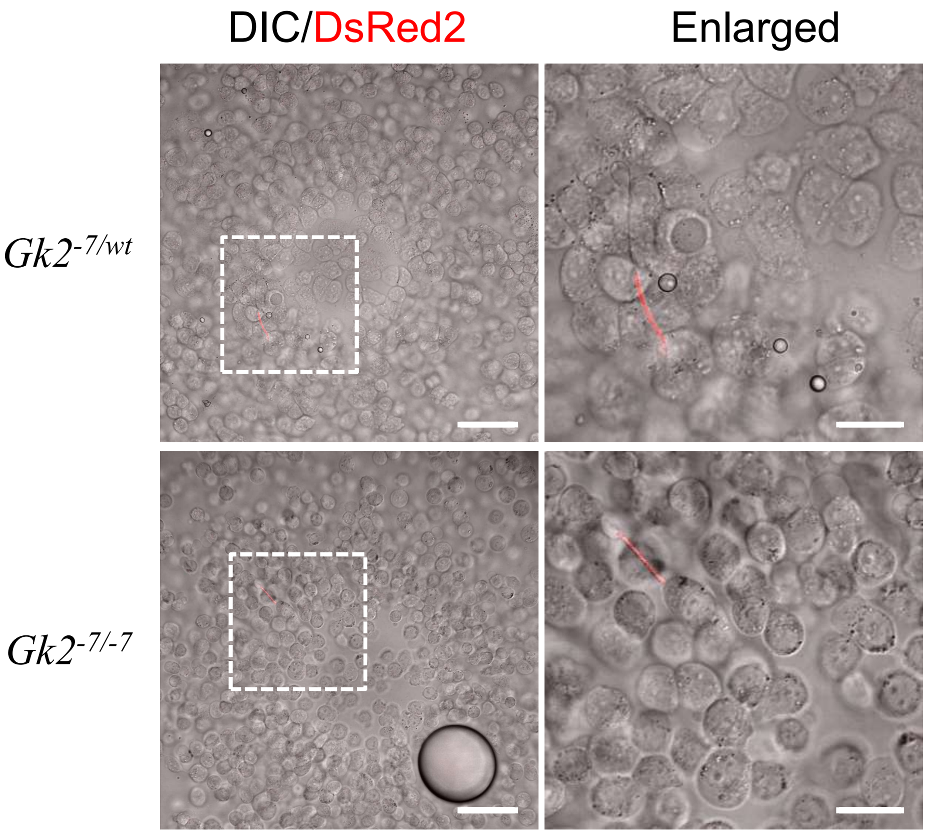

F

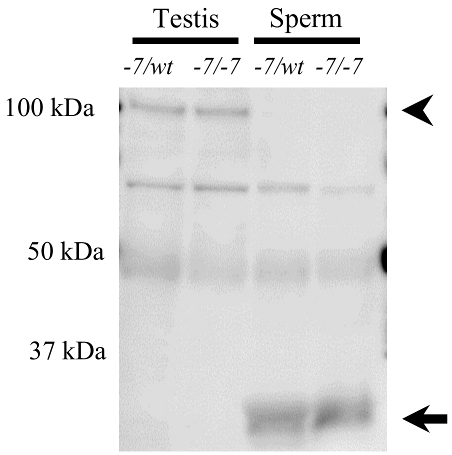

Fig. S2. *Gk2* KO spermatozoa show motility defects but not SEPT4 mislocalization or abnormal processing of ADAM3

(A) Immunofluorescence staining of SEPT4 in control and *Gk2* KO spermatozoa. *Gk2* KO mice with RBGS express both acrosome-targeted EGFP and mitochondria-targeted DsRed2. Spermatozoa collected from cauda epididymis of control and *Gk2* KO mice in the RBGS background were stained with antibodies against SEPT4 (green) and Hoechst 33342 (blue). Arrows show SEPT4 (annulus). Scale bars are 10 µm.

(B) Sperm motility of control and *Gk2* KO mice.

(C) The means of VAP; average path velocity, VSL; straight-line velocity and VCL; curvilinear velocity.

(D) Fertilization rate of in vitro fertilization (IVF) using control and *Gk2* KO spermatozoa. Three types of eggs (cumulus intact, cumulus free and zona free) were used for IVF.

(E) Cumulus cell penetration assay using control and *Gk2* KO spermatozoa. Left panels show the cumulus-oocyte complex which were incubated with control or *Gk2* KO spermatozoa. Right panels show enlarged images of the boxed areas. Scale bars are 50 µm (Left panels) and 20 µm (Right panels).

(F) Western-blot analysis of ADAM3 in both testis and cauda epididymal spermatozoa from control and *Gk2* KO mice. Arrowhead indicates ADAM3 precursor, and arrow shows the processed form of ADAM3.

* p<0.05, ** p<0.01, Student's t test, Error bars represent S.D.

Figure. S3 (Shimada *et al.*)

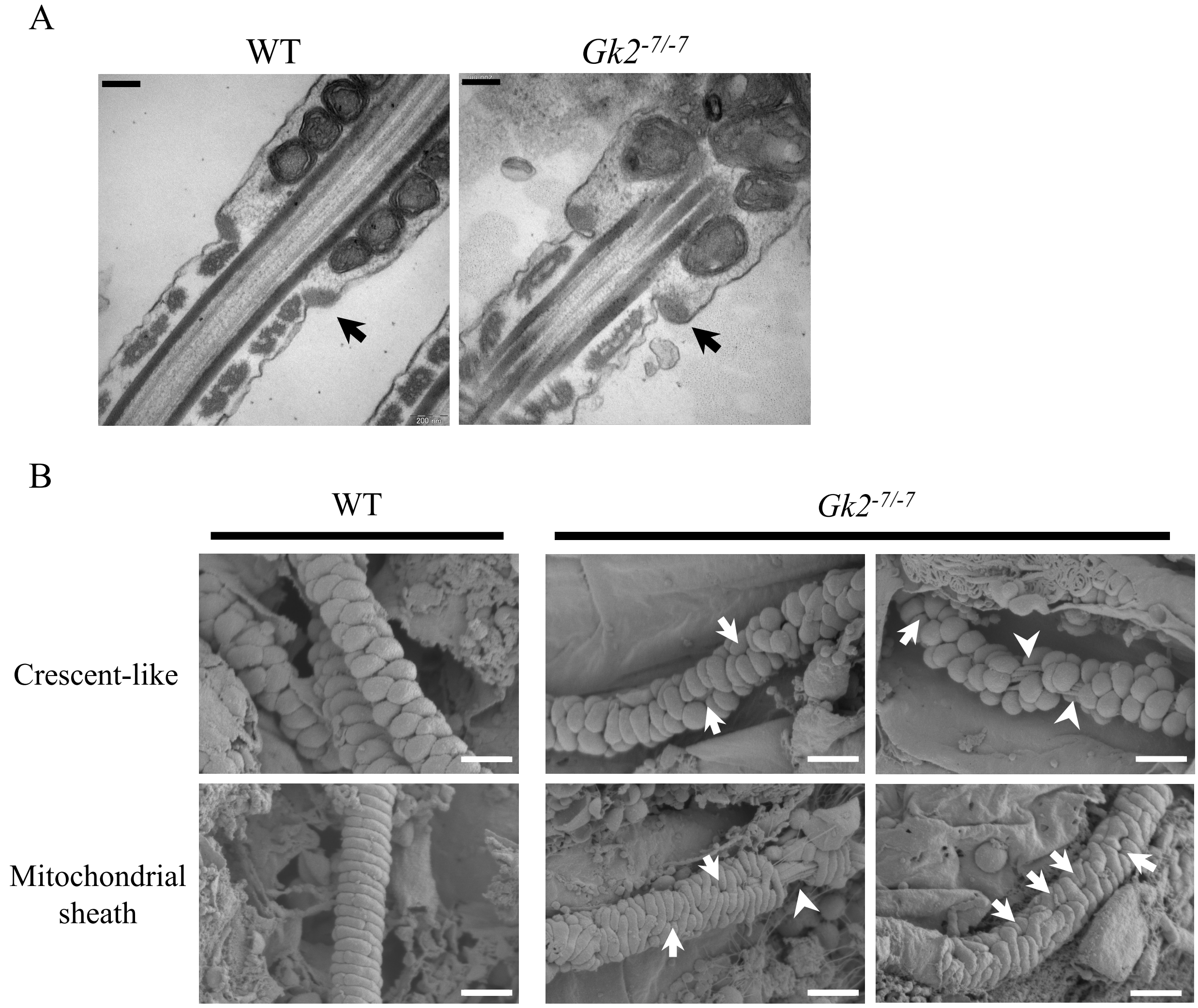

Fig. S3. Ultrastructure of *Gk2* deficient spermatids during spermatogenesis

(A) Annulus structure of spermatids at step 16 (stage VIII) observed by TEM. Arrows indicate annulus. Scale bars are 200 nm.

(B) Variety of *Gk2* deficient spermatids during spermatogenesis analyzed by SEM. Arrows show breaks of aligned mitochondria. Arrowheads show exposed outer dense fiber. Scale bars are 1 μm.
